# Effect of different packaging methods on the free amino acid profiles of the deep-water rose shrimp (*Parapenaeus longirostris*) during frozen storage

**DOI:** 10.3389/fnut.2022.955216

**Published:** 2022-07-27

**Authors:** Polina Rusanova, Gioacchino Bono, Manuela Dara, Francesca Falco, Vita Gancitano, Sabrina Lo Brutto, Charles Odilichukwu R. Okpala, Nilesh Prakash Nirmal, Federico Quattrocchi, Giacomo Sardo, Abdo Hassoun

**Affiliations:** ^1^Institute for Biological Resources and Marine Biotechnologies, National Research Council (IRBIM-CNR), Mazara del Vallo, Italy; ^2^Department of Biological, Geological and Environmental Sciences (BiGeA)–Marine Biology and Fisheries Laboratory of Fano (PU), University of Bologna, Bologna, Italy; ^3^Consorzio Nazionale Interuniversitario per le Scienze del Mare (CoNISMa), Rome, Italy; ^4^Department of Biological, Chemical and Pharmaceutical Sciences and Technologies (STEBICEF), University of Palermo, Palermo, Italy; ^5^Department of Functional Food Products Development, Faculty of Biotechnology and Food Science, Wroclaw University of Environmental and Life Sciences, Wroclaw, Poland; ^6^Institute of Nutrition, Mahidol University, Nakhon Pathom, Thailand; ^7^Sustainable AgriFoodtech Innovation & Research (SAFIR), Arras, France; ^8^Syrian Academic Expertise (SAE), Gaziantep, Turkey

**Keywords:** shrimp, free amino acids, modified atmosphere packaging, vacuum, sulfites, freezing shelf-life, seafood quality

## Abstract

The composition of free amino acids (FAAs) in seafood products contributes to characterizing their flavor, as well as freshness and quality during storage. Deep-water rose shrimps (*Parapenaues longirostris*, Lucas, 1846) (DWRS) are being increasingly harvested in the Mediterranean Sea, and the captured specimens are quickly frozen onboard fishing trawlers to preserve freshness and post-harvest quality. Here, we quantified the FAA profiles of DWRS packaged using five methods: (1) 100% N_2_; (2) vacuum; (3) 50% N_2_ + 50% CO_2_; (4) commercial anhydrous sodium sulfite; and (5) air (control). All samples were quickly frozen at −35°C and stored for 12 months at −18°C. Arginine (661 mg/100 g), proline (538 mg/100 g), and glycine (424 mg/100 g) were the most abundant FAAs, whereas the least abundant were tyrosine (67 mg/100 g), histidine (58 mg/100 g), and aspartic acid (34 mg/100 g). FAAs in all samples gradually (and significantly) increased in the first 6 to 8 months of storage, and then significantly decreased. The sodium sulfite treatment (Method 4) kept the initial FAA contents lower than the other treatments, due to the strong antioxidant action of sulfite agents. Interestingly, similar results were obtained for vacuum packaging (Method 2). Thus, combining frozen storage with vacuum packaging represents an alternative approach to chemical additives in shrimp/prawn processing to meet the increasing demand for high-quality seafood products with long shelf-life.

## Introduction

Free amino acids (FAAs) are among the most essential fractions of non-protein nitrogen compounds found in the tissues and muscles of several seafood products (1–3). Some FAAs, such as alanine, glycine, lysine, and taurine occur at relatively high concentrations in fishery products and are the basis for a balanced healthy diet (4). Many important fractions of non-protein nitrogenous compounds could be used as indicators of food product spoilage, as they are the precursors of biogenic amines (5). FAAs also determine the sweetness, sourness, bitterness, and umami taste of fish products (3, 6). For instance, glycine, alanine, and glutamic acid are associated with the typical “umami” taste in crustacean products (1). In particular, FAAs can interact with reducing sugars, which is demonstrated *via* the Maillard reaction or non-enzymatic browning in seafood products, altering aroma, color, and taste (3). The composition of FAAs in seafood products inevitably changes during storage as a function of food packaging and processing technologies (3).

In the Mediterranean region, after harvesting, shrimp products are preserved directly onboard fishing trawlers using air blast freezing techniques (at −35°C), followed by storage at −18°C in a static freezer (7). Although the freezing process prevents bacterial activity, biochemical processes continue to take place at a very slow rate during storage, irreversibly altering the freshness and flavor (8). In addition, the preservative success of freezing is affected by the size and muscle structure of fishery products (7, 9). Bono et al. (7, 10, 11) showed that the ability of MAPs to prevent fishery products from deteriorating increases when they are combined with other preservation methods, such as freezing. For instance, Bono et al. (10) showed that lipid oxidation and volatile amines were significantly reduced in DWRS when MAP was combined with frozen storage. Yet, the freshness of a fish product varies noticeably depending on packaging method (N_2_, CO_2_, or vacuum package), storage temperature, and species. Therefore, it is important to quantify FAAs from a production perspective of biogenic amines, due to the capacity of MAP/vacuum package to preserve the FAA profile of seafood products (7), as well as documented changes in FAA concentrations during processing and storage (12).

In countries bordering the Mediterranean Sea (i.e., Italy, France, Spain, Algeria, Tunisia, Greece, and Turkey), DWRS are one of the most highly harvested crustaceans (13–15). DWRS are usually caught by bottom trawling vessels, and are quickly frozen onboard to preserve the high quality of the fresh harvest. However, dark discoloration that arises post mortem makes the shrimp unacceptable for purchase by consumers (16). Previous studies demonstrated that post mortem discoloration could be prevented by pretreating DWRS with various chemical approaches, such as those using chemical antioxidants (e.g., resorcinol, sulfites), melanosis-inhibiting formulations, phenolic extract derived from olive vegetation water, or applying non-thermal processes, such as MAP combined with frozen storage (10, 17–19). Quantifying FAAs in fishery products could advance our current understanding of various autolytic processes, as well as muscle degradation, which decisively influences FAA evolution (20, 21). Investigating how FAA levels in DWRS change under frozen storage combined with MAP/vacuum could help promote the use of this approach by the fishermen and associated stakeholders in the Mediterranean basin. It might also provide a baseline for assessing general risks to health from consuming seafood products packaged under different methods and conditions.

Thus, here, we aimed to determine FAA content in DWRS after harvesting and to assess the impact of five different packaging methods, followed by frozen storage at −18°C for 12 months on FAA profiles.

## Materials and methods

### Overview of the experimental program

**Figure 1:**
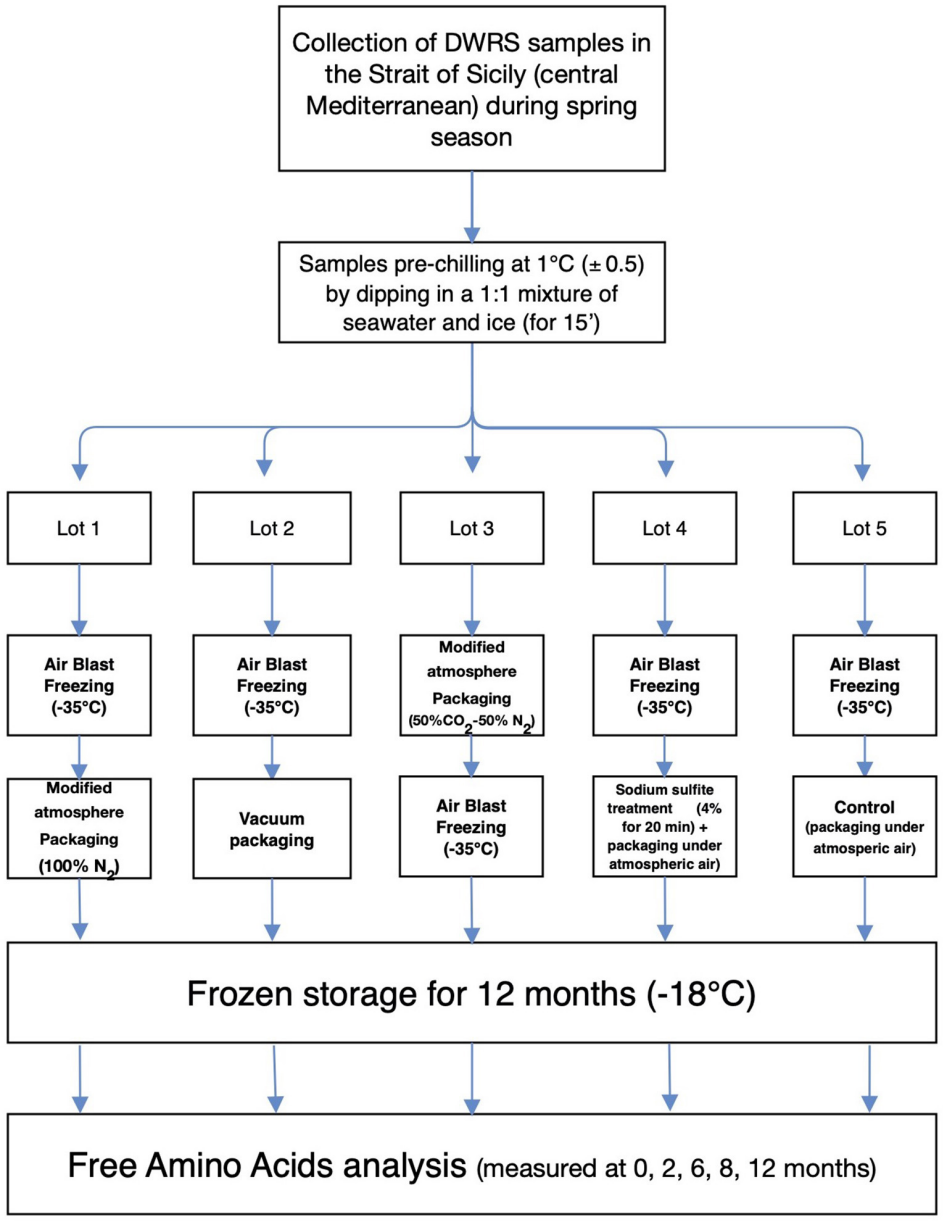
Schematic representation showing an overview of the experimental program, starting from the harvesting of DWRS samples by bottom trawl vessels to the analytical determination of FAAs.

An overview of the experimental program is presented in Figure 1. This schematic representation depicts the major stages of this work, including harvesting DWRS samples by bottom trawl vessels, onboard collection, handling, packaging protocols, and analytical determination of FAAs. FAA analysis was initiated on day 6 after the catch and onboard packaging of DWRS, due to the time required for landing and transfer of samples to the laboratory. Thus, “time zero” captured this delay used in the following sections and graphs. Before laboratory analyses, DWRS samples were thawed, beheaded, and peeled. All chemicals and reagents employed were of analytical grade.

### Onboard handling, packaging, and frozen storage of shrimp

#### Handling and packaging

DWRS samples were processed directly onboard a shrimp trawler, according to the method described by Bono et al. (7, 10) with minor modifications. A bottom trawler equipped with a semi-automatic MAP system (Mondini, Brescia, Italy) was used to collect 50 kg of shrimp during a single haul (to remove any bias caused by sampling time and/or fishing operation). Shrimp had an average carapace length of 22 ± 5 mm. Shrimp were washed in flowing seawater and pre-chilled (1 ± 0.5°C) within 1 h of capture, by dipping them in a 1:1 mixture of seawater and ice. After approximately 15 min, when the core temperature of the shrimp was equal to the temperature of the ice-seawater mixture, the ice water was drained from the shrimp, and the shrimp were randomly separated into five lots for the five treatment/packaging protocols (Figure 1):

- Lot 1 was quickly frozen in an air blast freezer room at −35°C (air speed of 4–5 m/s). When the water at the thermal center of the DWRS samples became ice, they were packaged under 100% N_2_ gas using the semi-automatic MAP system;
- Lot 2 was frozen in air in the blast freezer room at −35°C (similar to Lot 1). It was then vacuum-packaged using the semi-automatic MAP system;
- Lot 3 was packaged under 50% N_2_ + 50% CO_2_ gas mixture to allow the full dissolution of CO_2_ in the shrimp tissue (22), and was then frozen in the air blast freezer room at −35°C;
- Lot 4 was dipped in seawater solution (4% w/v) of commercial anhydrous sodium sulfite (shrimp-to-dipping solution ratio of 1:4), according to the preservation techniques and materials usually employed by Mediterranean fleet crews. After 20 min dipping, shrimp were dried, frozen in the air blast freezer room at −35°C, and finally packed under atmospheric air using the semi-automatic MAP system;
- Lot 5 was frozen in the air blast freezer room at −35°C with no treatment, packed under atmospheric air using the semi-automatic MAP system and served as the control for the current study.

#### Packaging materials

DWRS samples were packaged using the method previously described by Bono et al. (7). Transparent A.PET/EVOH/PE barrier bags (bag size: 290 × 200 mm; bag volume: 1.8 L; laminate density: 1.39 g/cm^3^; thickness: 500 μm) (Arcoplastica Srl, Andezeno, Italy) were used for packaging, and were manufactured with oxygen (O_2_) and water vapor permeabilities of 1.8 cm^3^ m^−2^ day^−1^ atm^−1^ and 4 g m^−2^ day^−1^, respectively. Packaging bags were heat-sealed by a semi-automatic packaging machine (Mondini S.p.A., Brescia, Italy), which employed a multiflex OPP/EVOH/PE (Cryovac, Sealed Air Corp., Italy) film (weight: 72 g m^2^; thickness: 75 μm) that operated O_2_, CO_2_, and water vapor permeabilities of 3 cm^3^ m^−2^ day^−1^ atm^−1^, 10 cm^3^ m^−2^ day^−1^ atm^−1^ and 3 g m^−2^ day^−1^, respectively. For MAP samples, a gas-to-product ratio (v/w) of 2.25:1 was applied. An additional anti-pinhole lamina (weight: 340 g/m^−2^; thickness: 250 μm) was inserted in the headspace, which helped to minimize damage to the multiflex film by shrimp horns.

#### Frozen storage

When all packaging operations onboard were complete, all packaged DWRS samples were subjected to frozen storage (−18°C) for 12 months. According to Blond and le Meste (23), this is the optimum storage temperature when considering both the financial costs of freezing and shelf-life of frozen foods. The frozen storage of DWRS samples adhered consistently to widely recognized storage regulations in Europe and other countries globally, particularly for the temperatures of frozen foods (7).

### Determination of FAA profiles

The FAAs were analyzed, from extraction to chromatography, according to a previously described method (7, 24), with minor modifications. Ten grams of shrimp and 40 mL extracting solvent (75% methanol in distilled deionized water) were homogenated, transferred to a 100 mL volumetric flask, and stored for 60 min at 4°C. The contents of the flask were transferred to a 50 mL centrifuge tube and centrifuged at 15,000 rpm for 40 min at 4°C. The supernatant was filtered on a PTFE 0.2 μm filter membrane (Gelman Sciences), before derivatization. By way of o-phthaldialdehyde (OPA) pre-column derivatization, the OPA Thiol Reagent (OPT) was prepared 24 h before use by dissolving 27 mg of o-phthaldialdehyde in 500 μL absolute alcohol. Then, 5 mL of 0.1 M sodium tetraborate (Na_2_B_4_O_7_,10H_2_O) (pH 9.5) were added, followed by 50 μL mercaptoethanol, which were mixed and stored in the dark. The amino acid standard stock solution was prepared by dissolving the equivalent of 2,500 nmol of each amino acid in 0.05 M NaH_2_PO_4_ buffer (pH 5.5), which was then diluted for the calibration curve. Then, 400 μL of OPT was added to 100 μL of the amino acid standard or 189 diluted sample supernatant under High Pressure Liquid Chromatography (HPLC), and the sample was manually injected onto the column of the HPLC system. Mobile phase A was made up of 0.05 M sodium phosphate buffer (pH 5.5), methanol, and tetrahydrofuram (THF) (80: 19:1). Mobile phase B was made up of 80% methanol and 20% of the 0.05 M NaH_2_PO_4_ buffer. The pH of the phosphate buffer was adjusted to 5.5. The mobile phases were filtered through 0.2 μm filter membranes (Gelman Sciences, Ann Arbor, MI) and were degassed by vacuum for 5 min. The HPLC column was an Ultrasphere ODS with 5 μm particle size, 4.6 mm × 25 cm (Beckman Instruments, Inc., Fullerton, CA). The elution gradient was generated using an Elite LaChrom equipped with a L-7100 pump (LaChrom, Hitachi), oven L-7350 (LaChrom, Merck), programmable fluorescence detector, which had an excitation monochromator setting of 330 nm and emission cut-off filter of 418 nm. Chromatographic data were processed using software EZChrom Elite (Agilent Tech., Santa Clara, CA 95051, USA). The FAA results are presented as the mean of four replicates ± standard deviation and expressed in mg/100 g.

### Statistical analyses

For each FAA, we tested the differences between treatments and the existence of linear and simple polynomial trend as function of the storage time. Ordinary least square models were used introducing the treatment as factor (fixed with 5 levels) and time as continuous variable. Models were fitted in which the highest order polynomial term can be linear, quadratic, or cubic function of time. This analysis, also called trend analysis, is basically an extended ANCOVA which allows us to model quantitative predictors with higher-order polynomials. Fitting a polynomial allowed us to express the impact of the continuous predictor (time in this case) on the response to separately evaluate the contributions of linear and non-linear components of the polynomial. The best formulation was chosen using the small-sample equivalent Akaike Information Criterion (AICc). A Tukey test was used to perform pairwise comparisons between levels of the factor treatment when it was significant in the regression analysis.

We used heat maps to visualize, graphically and simultaneously, clusters of FAA concentrations across treatments and storage time. Hierarchical clustering on the Euclidean dissimilarity matrix was first performed on both the treatments and time storage, using the complete linkage method in which the distances between two clusters are defined as the maximum value of all pairwise distances between elements in of different clusters. Then, it was visualized by re-ordering the observations based on their similarity according to the hierarchical clustering results. The heat maps were constructed by using the “heatmap” functions in the “stats” package which is part of R statistical software (37).

## Results and discussion

### Overall snapshot of FAAs

**Figure 2:**
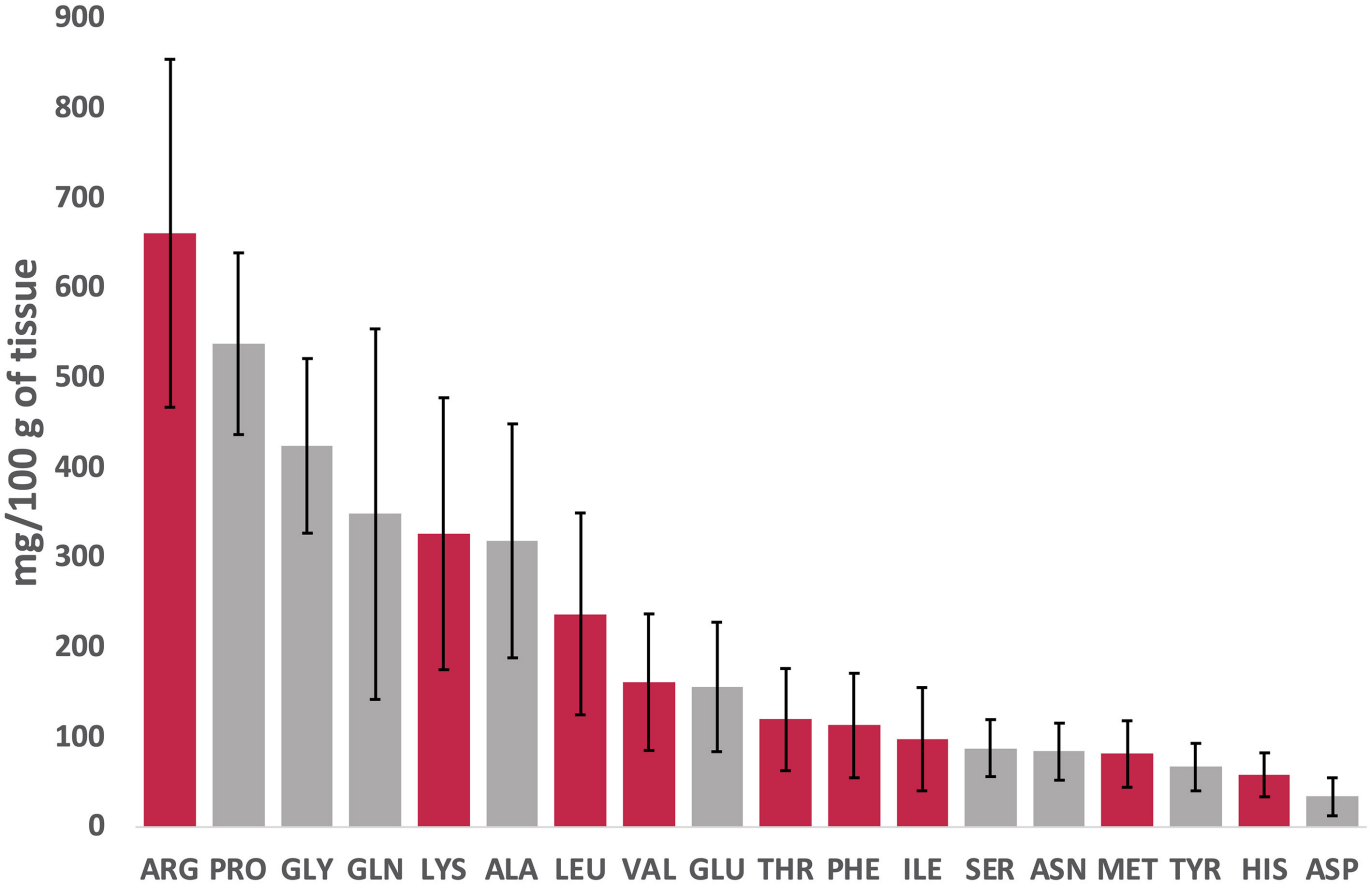
Overview of free amino acids (FAAs) concentrations (essential, red; non-essential, gray) in DWRS arranged in decreasing order (left to right), regardless of the effect of storage time (12 months at −18°C) or packaging method.

Figure 2 presents a snapshot of each FAA (essential and non-essential AAs are presented in red and gray, respectively) in DWRS caught in the Strait of Sicily during spring season. The data are arranged in decreasing order (from left to right), regardless of the effects of storage time (12 months at −18°C) and the five packaging methods.

Three FAAs constituted more than 40% of total FAAs (Figure 2). Arginine was the most prominent (661 mg/100 g; 17% of total), followed by proline (538 mg/100 g equal to 14%) and glycine (424 mg/100 g equal to 11%). Aspartic acid, histidine, and tyrosine had the least concentrations. The high levels of glycine and arginine detected corroborated that reported for the Norway lobster *Nephrops norvegicus* (Linnaeus 1758) in the same region (11), and prawns (25). The ratio of essential amino acids (EAAs) to non-essential amino acids (NEAAs) was 0.9 (data not shown), which was higher than that reported by Iwasaki and Harada (26) for many other seafood products (0.7). According to Gómez-Limia et al. (3), the amount of EAAs is an important factor that affects the nutritional value of proteins.

High levels of typical (sweet) FAAs such as arginine, proline, and glycine (27) detected in this study might be associated with the high acceptability and palatability, particularly for shrimp and lobsters. In addition, high quantities of FAAs detected in DWRS (such as glutamic acid, glutamine, lysine and arginine) could exhibit anxiolytic-like and/or antidepressant-like activity, and might reduce corticosterone and cortisol levels in stressed animals (28). There was also a noticeable variation for given FAAs (which was more marked in some), which might be associated with storage time (one year) and/or packaging method.

**Figure 3:**
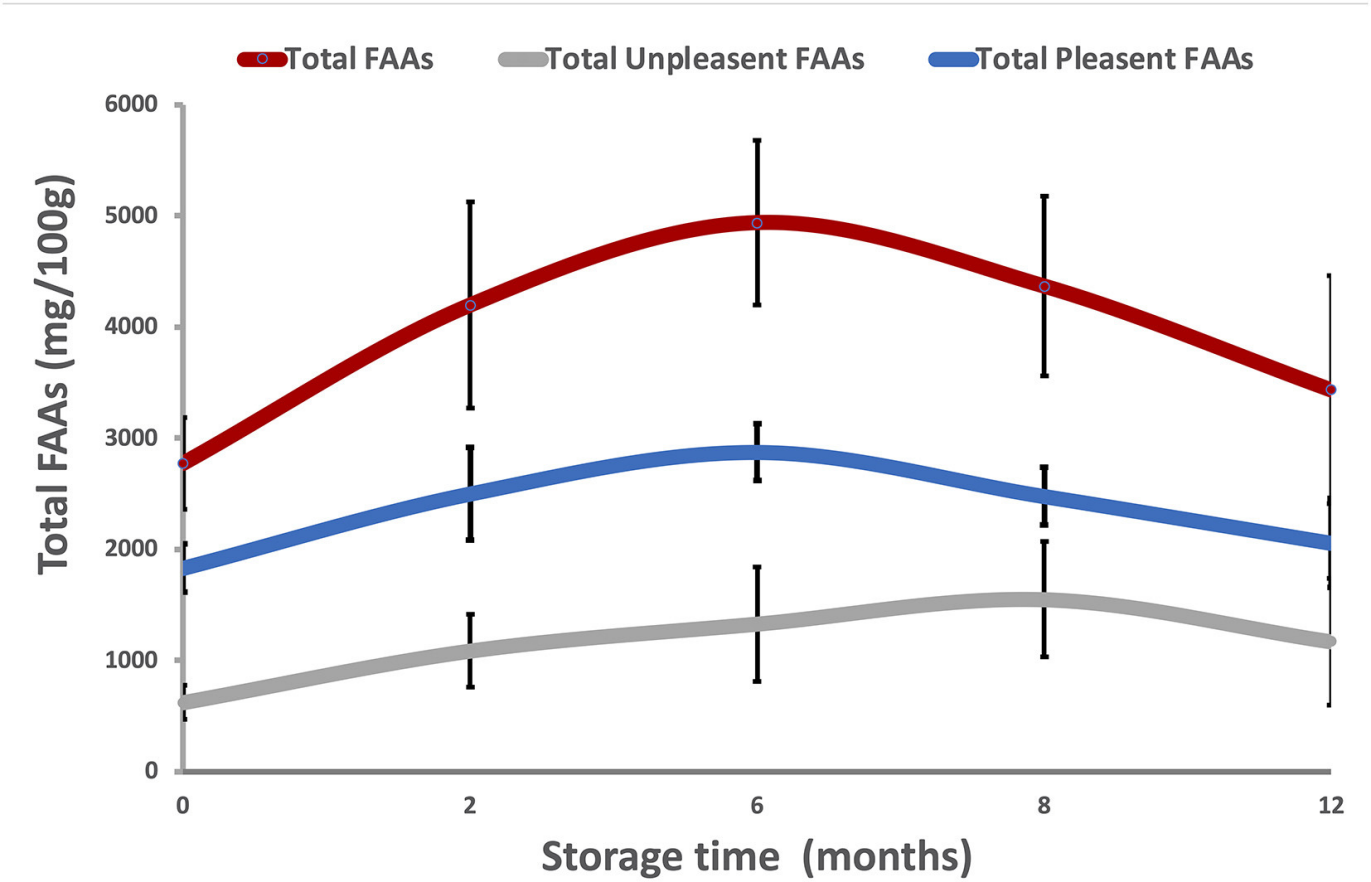
Trends in total FAAs (red), pleasant FAAs (PFAAs, blue), and unpleasant FAAs (UPFAAs, gray) in DWRP taking frozen storage time into account (12 months at −18°C), regardless of the effects of packaging methods.

According to Chen and Zhang (27) and Dai et al. (29), FAAs can be grouped into two main categories: pleasant FAAs (PFAAs), including threonine, glutamic acid, aspartic acid, alanine, glycine, serine, proline and arginine, and unpleasant FAAs (UPFAAs), including leucine, phenylalanine, isoleucine, lysine, valine, methionine, tyrosine, histidine and cysteine. Based on this classification, Figure 3 shows the trends in total FAAs, total PFAAs, and total UPFAAs in DWRP after 12 months of storage at −18°C, regardless of the effects of packaging. The total FAA content of all five packaging methods increased over the treatment period (start: 2,781 mg/100 g; after 6 months storage: 4,937 mg/100 g). Then, the total FAAs content decreased slightly until the end of storage (12 months; mean 3,535 mg/100 g). The increase of total FAAs during the first/eight months of storage might be associated with the degradation of proteins *via* endogenous protease activity (30). Over the 12-month period, mean PFAA content ranged between 1,832 and 2,058 mg/100 g in the five groups. Similarly, over the 12 month period, total UPFAA concentrations ranged between 624 and 1,171 mg/100 g. Comparing our results with those in existing literature, the zero timepoint FAA observation in our study was about 30% higher than that previously recorded for the same species (31). This difference might be explained by the 6 days of time-lapse between the shrimps catch and the FAAs analysis or attributed to geographical factors (i.e., different fishing areas; Central Mediterranean vs. East Atlantic Ocean) or biological factors (i.e., trophic conditions, catch period, size and developmental stage of specimens, quantity and distribution of lipids in muscle, especially in lean specimens).

### Changes to FAAs during frozen storage time

**Figure 4:**
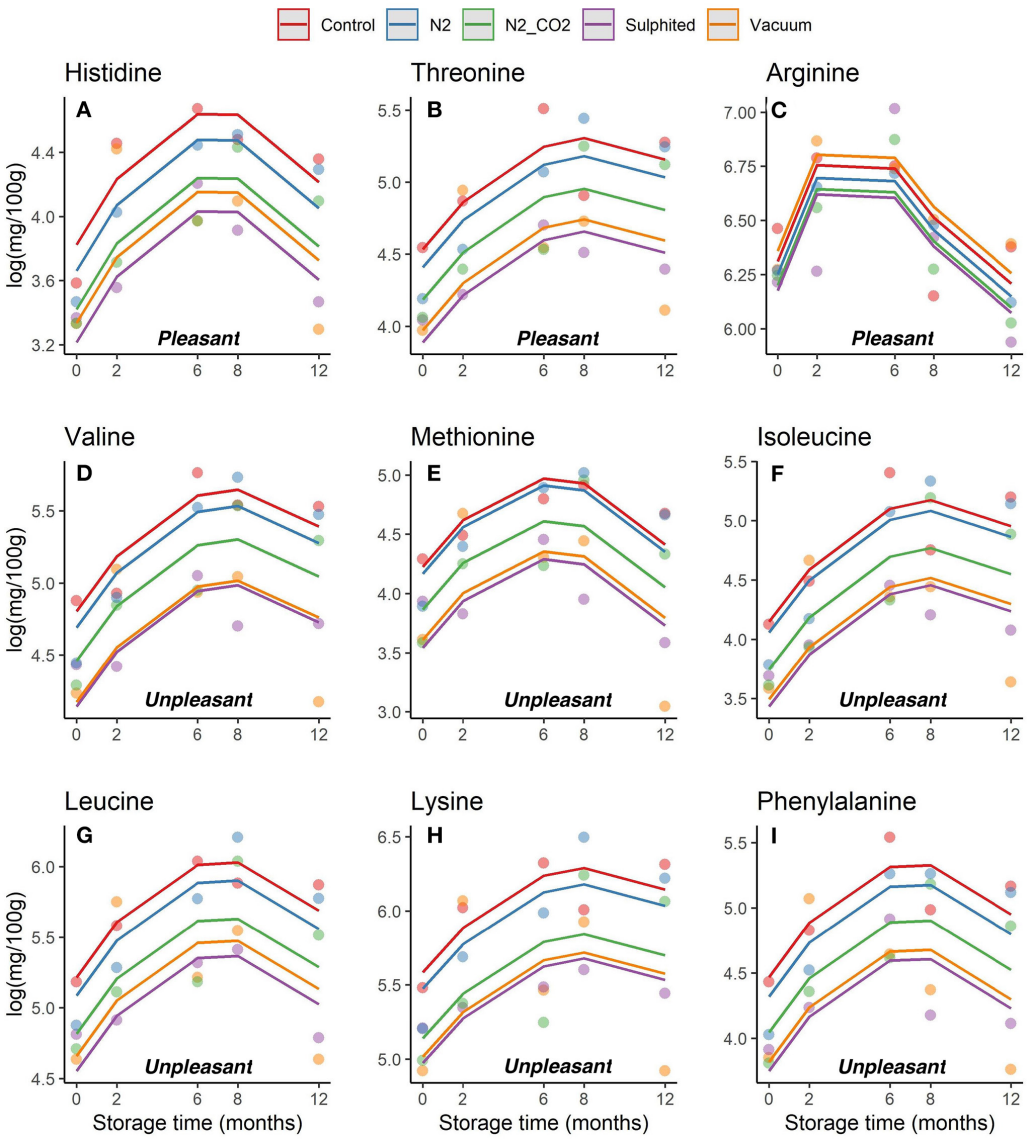
**(A–I)** Trends obtained for each essential FAA in DWRS samples in relation to the packaging method over 12 months of frozen storage. FAAs were grouped as pleasant and unpleasant.

**Figure 5:**
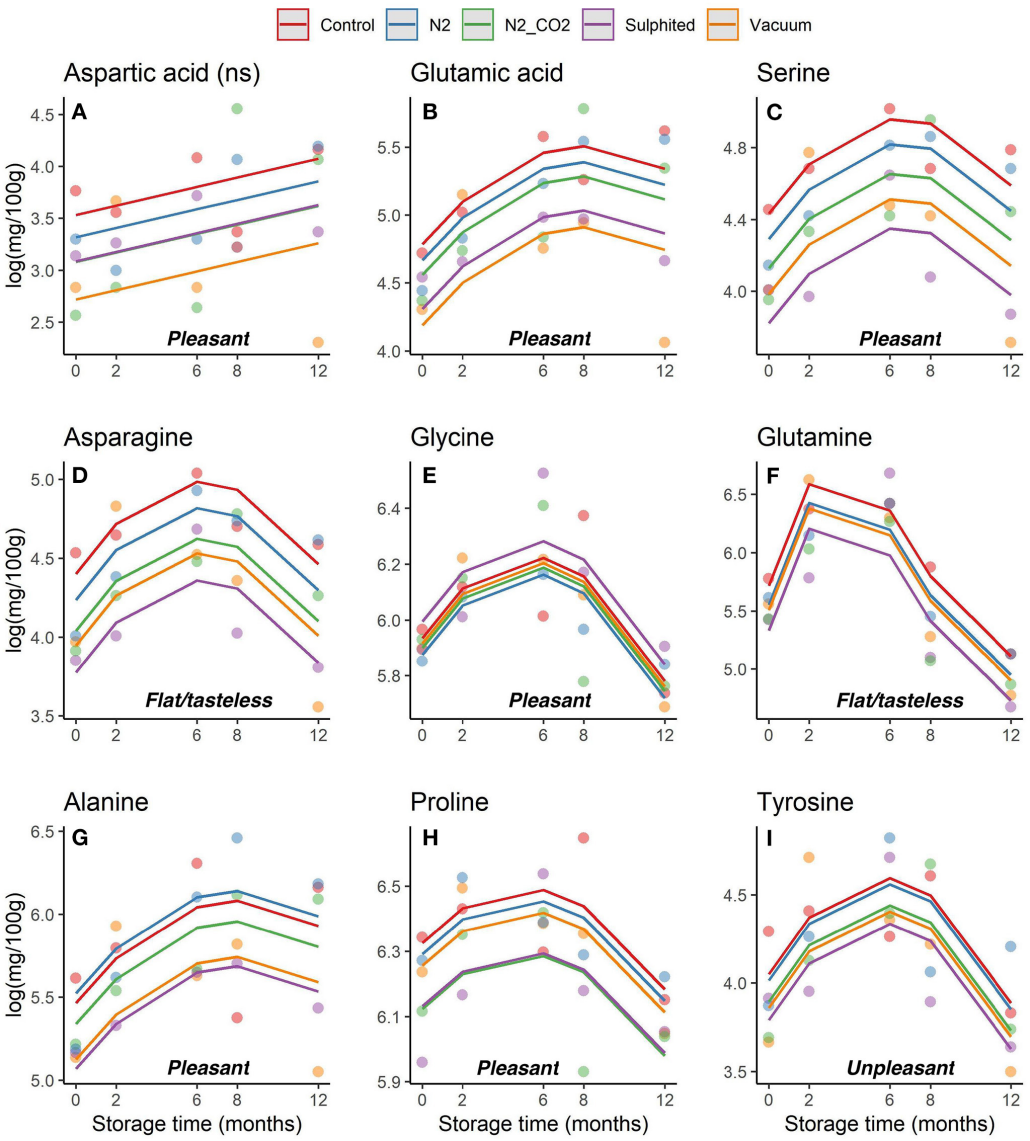
**(A–I)** Trends obtained for each non-essential FAA in DWRS samples in relation to the packaging method over 12 months of frozen storage. FAAs were grouped as pleasant, unpleasant, and flat/tasteless.

Trends in the concentrations of each essential and non-essential FAA (pleasant, unpleasant and flat/tasteless) in DWRS samples per packaging/treatment method and frozen storage time were evaluated (Figures 4, 5, respectively). The concentrations of each FAA differed with packaging method, several of which depicted diverse non- and bow-like shapes. On day 6 after catch (time 0 on the graph), the concentrations of some FAAs already exhibited significant differences among the five packaging methods, especially for both sulfited and vacuum lots when compared to N_2_, N_2_/CO_2_, and control. Thus, FAAs appear to change immediately after the catch, with preservative treatment having a strong effect.

**Figure 6:**
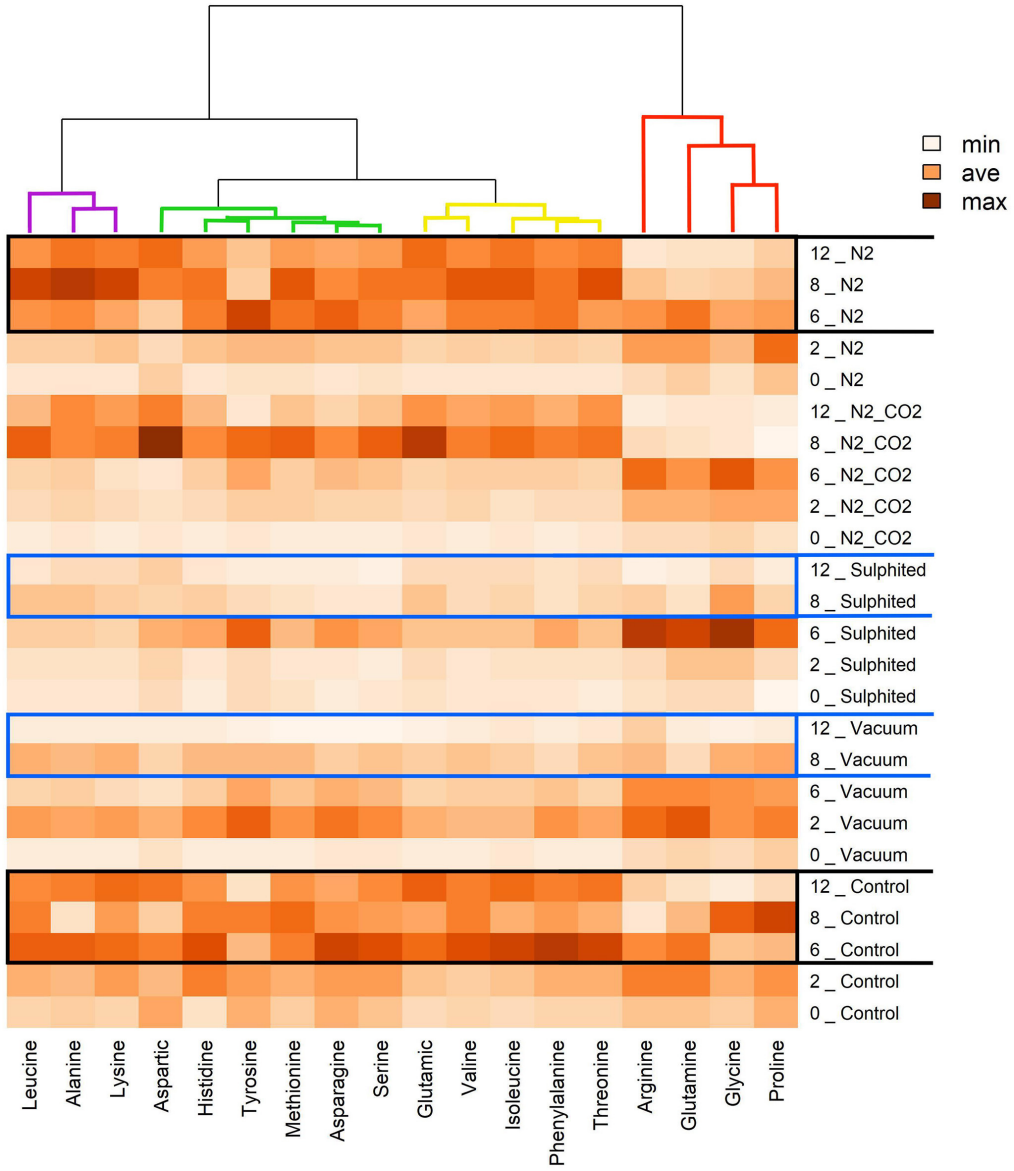
Heat map visualization and clustering results of FAAs of deep water rose shrimps during frozen storage. The heat map was generated using hierarchical clustering analysis to identify the color intensity for each FAA (minimum intensity, no color; average intensity, yellow; maximum intensity, brown). Values adjacent to packaging methods represent frozen storage time in months.

**Figure 7:**
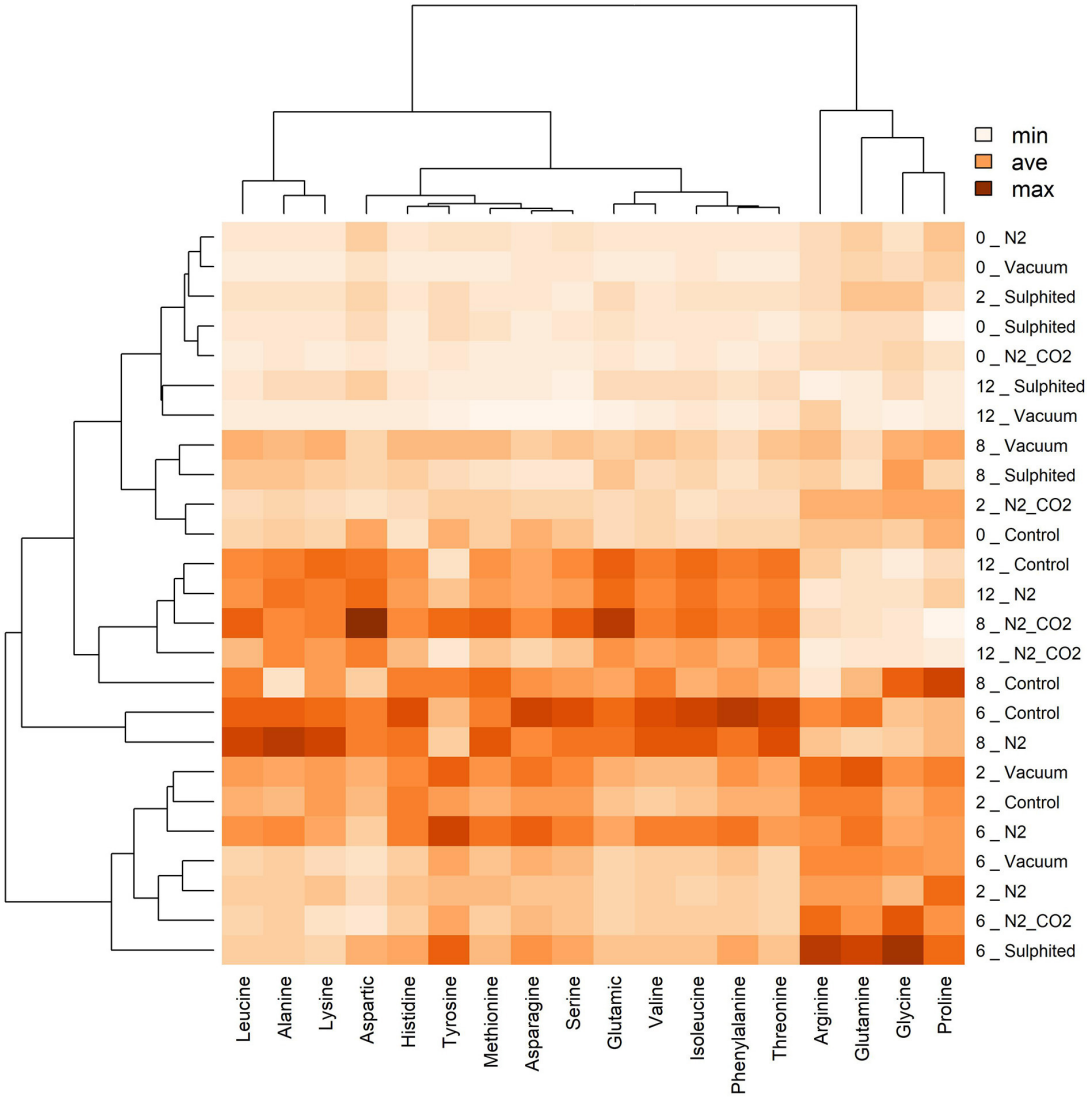
Heat map visualization and clustering results of FAAs in deep water rose shrimps during frozen storage, regardless of the effects packaging method (y-axis). Similar to Figure 6, the heat map was generated using hierarchical clustering analysis to identify the color intensity of each FAA (minimum intensity, no color; average intensity, yellow; maximum intensity, brown). Values adjacent to packaging methods represent frozen storage time in months.

During the first 6–8 months of frozen storage time, all FAAs (essential/non-essential, pleasant/unpleasant), except aspartic acid, showed a gradual significant increase, and then declined at 8–12 months (*p* < 0,05). Pleasant FAAs that constituted over 40% of total FAAs (i.e., arginine (Figure 4C), proline (Figure 5H), and glycine (Figure 5E) exhibited a similar pattern, rising until the sixth month, and declining until the 12^th^ month of frozen storage, with ultimate values being slightly lower than those detected at zero time. The similarity among arginine, proline, glycine, and glutamine was also confirmed by the heat-map coupled with cluster analysis depicted in Figure 6 in which the hierarchical clustering was applied only on the FAAs. Proline appeared to be strongly correlated with glycine, while glutamine (one of flat/tasteless FAAs) was correlated with arginine (red cluster). On the other hand, Figure 7 showed the hierarchical clustering applied on both the treatments and FAAs.

When considering the effect of treatments on each FAA, the cluster analysis also highlighted three other groupings. Threonine, phenylalanine, isoleucine, valine, and glutamic acid were clustered together to form a second group (yellow lines). Serine, asparagine, methionine, tyrosine, histidine, and aspartic acid (green lines) were clustered together to form a third group. Lysine, alanine, and leucine (violet lines) were clustered together to form a fourth group.

Hong et al. (32) found that the loss of quality is generally accompanied by an increase in glycine, whereas higher glycine levels denote high quality and acceptability of fish products. Certain FAAs (such as arginine, proline, and glycine) have important roles in the freshness and palatability of fish and shellfish muscle. Our results showed that DWRS samples remained in good condition throughout the 12 months of storage. The best condition for DWRS samples was reached at the sixth month, when arginine, proline, and glycine approached maximum values (>500 mg/100 g) (27, 33).

Protein degradation by endogenous protease should also be evaluated when exploring shelf-life during frozen storage, as it caused FAAs to increase, particularly at 6–8 months of storage. Furthermore, protein degradation is impacted by the rate of freezing, effect of ice crystals, and packaging method. The general reduction in FAAs observed in the last 3 months, reflected that also recorded for Norway lobster by Bono et al. (11). This phenomenon might be attributed to decarboxylation by decarboxylase enzymes, and the consequent formation of breakdown products such as biogenic amines (34, 35) that can cause relevant modifications in the sensory properties, nutritional and safety quality of seafood products with negative effects on consumers health. Indeed, consuming seafood products with high levels of histamine, tyramine and cadaverine (formed by decarboxylation of histidine, tyrosine and lysine, respectively) can produce some acute forms of urticaria, vomiting and diarrhea, as well as could be involved in more serious complications affecting the nervous and vascular system, or become potential precursors of carcinogens (3).

### Changes to FAAs under the five packaging methods

For all five tested packaging methods, the levels of arginine, proline, and glycine (first, second, and third most abundance FAAs, respectively), as well as glutamic acid, glutamine, alanine, and tyrosine were similar (Figures 4, 5; Supplementary table 1). A similar result was obtained by Bono et al. (7) for Giant red shrimp *Aristeomorpha foliacea* (Risso, 1827) caught in the same fishing area. On the other hand, Supplementary table 2 shows the result of the Tukey test used to perform a paired comparison between levels of the factor treatment when it was significant in the regression analysis.

The sulfiting treatment maintained the initial state of FAAs lower than the four other preservation methods. This result was obtained for most detected FAAs, and can be attributed to the strong antioxidant action of sulfite additives (36). Vacuum packed samples had the most similar results to sulfited samples for FAAs. Heat map analysis also confirmed the stability of FAAs with sulfiting agents during storage (Figure 6). Furthermore, heat maps confirmed our second unexpected finding on the stability of vacuum FAAs, especially at 8–12 months storage (Figure 6, two blue rectangles for sulfited and vacuum methods). In contrast, the heatmap showed that nitrogen preservation caused FAAs to change in a similar way to the control sample, especially in the last 6 months (intense coloring; two black rectangles Figure 6).

The good performance of FAAs in samples packed under vacuum to resemble those of sulfite-treated samples required further considerations. This result was novel, and was not obtained in the two previous analogous studies on Giant red shrimp (7) and Norway lobster (11) caught in the same geographical region. If the high performance of vacuum samples was due to the almost total absence of oxygen in the headspace (which is directly involved in the oxidative denaturation/deamination of proteins and FAAs), we should have obtained similar results for samples preserved in N_2_ (100%), as they were also packaged in anoxic atmosphere. Indeed, in our similar study on FAAs in giant red shrimp (7), 100% N_2_-treated group appeared with improved individual FAAs compared with other treatments.

As for FAAs in shrimp samples preserved in N_2_/CO_2_ (whose content was slightly higher compared to sulfited/vacuum, but lower compared to N_2_ and control samples), this result might be due to the positive acidification effect of CO_2_ on muscle tissue.

However, the FAA content of our samples was higher for N_2_ (100%) compared to N_2_/CO_2_ (50%-50%), and was similar to the control sample. As equivalent studies are currently not available in the published literature, more research on this phenomenon is required to draw conclusions on the evolution of FAAs in frozen seafood products.

## Conclusions

In this current study, changes in FAA profiles of post-harvest DWRS subjected to five different MAP methods, quickly frozen at −35°C, and thereafter kept for 12 months in frozen storage (−18°C) were investigated. Although the concentrations of FAAs differed with packaging and treatment methods, they appeared very high compared to the control. Furthermore, the ratio between essential vs. non-essential amino acids, as well as pleasant vs. unpleasant FFAs, was, interestingly, unbalanced in favor of the first ones (i.e., essential and pleasant amino acids). Additionally, regardless of the effects of treatment/packaging methods, the typical (sweet) FAAs arginine, proline and glycine constituted more than 40% of total FAAs. This might be associated with the high acceptability and palatability that characterize this valuable DWRS product. More so, the sulfiting treatment maintained the initial state of FAAs lower than the four other preservation methods. Overall, the frozen storage combined with MAP continues to be a promising alternative to chemical additives in shrimp/prawn processing, which not only could help sustain the FAAs as this current work has demonstrated, but more so, would help cater for increasing global demand for high-quality/lengthened shelf seafood products. Given the findings of this current work, the direction of future studies should be to subject the DWRS under the same treatment conditions to further shelf-life studies, especially biogenic amines and sensorial analysis.

## Data availability statement

The raw data supporting the conclusions of this article will be made available by the authors, without undue reservation.

## Author contributions

GB and CO provided conceptualization and study design. GB, PR, FQ, MD, FF, VG, and GS conducted experiments and performed data analysis. GB, PR, and MD drafted the initial manuscript. NN, SL, and AH critically reviewed and revised the manuscript. All authors approved the final manuscript.

## Conflict of interest

The authors declare that the research was conducted in the absence of any commercial or financial relationships that could be construed as a potential conflict of interest.

## Publisher's note

All claims expressed in this article are solely those of the authors and do not necessarily represent those of their affiliated organizations, or those of the publisher, the editors and the reviewers. Any product that may be evaluated in this article, or claim that may be made by its manufacturer, is not guaranteed or endorsed by the publisher.
